#  Human *Dirofilaria repens* Infection in Romania: A Case Report

**DOI:** 10.1155/2012/472976

**Published:** 2012-02-07

**Authors:** Ioana Popescu, Irina Tudose, Paul Racz, Birgit Muntau, Calin Giurcaneanu, Sven Poppert

**Affiliations:** ^1^Dermatology Department, Elias Emergency University Hospital, 011461 Bucharest, Romania; ^2^Histopathology Department, Elias Emergency University Hospital, 011461 Bucharest, Romania; ^3^Department of Clinical Diagnostics, Bernhard Nocht Institute for Tropical Medicine, Bernhard-Nocht-Stra*β*e 74, 20359 Hamburg, Germany

## Abstract

Human dirofilariasis is a zoonotic infectious disease caused by the filarial nematodes of dogs *Dirofilaria repens* and *Dirofilaria immitis*. Depending on the species involved, human infections usually manifest as one cutaneous or visceral larva migrans that forms a painless nodule in the later course of disease. Dirofilariae are endemic in the Mediterranean, particularly in Italy. They are considered as emerging pathogens currently increasing their geographical range. We present one of the few known cases of human dirofilariasis caused by *D. repens* in Romania. The patient developed unusual and severe clinical manifestations that mimicked pathological conditions like cellulitis or deep venous thrombosis.

## 1. Introduction

Human dirofilariasis is a zoonotic infectious disease caused by parasites of the genus *Dirofilaria* [1–3]. Dirofilariae are a group of filarial nematodes infecting various carnivores as definitive hosts. The adult dirofilariae release microfilaria into the host's blood, which may be taken up by various mosquitoes that serve as intermediate hosts and transmit the disease. Humans may be infected as aberrant hosts, mainly by *Dirofilaria (D.) immitis* and *D. repens. *The definitive hosts of *D. repens *and *D. immitis* are dogs, but other animals have also been reported as reservoirs of the disease (cats, wolves, bears, foxes, etc.) [1, 3]. In human infections usually just one larva develops, which does not reach fertility [2, 3]. The larva wanders through the human body and finally forms a nodule. *D. repens* usually resides subcutaneously, while *D. immitis* frequently ends up in the human lung. In most cases parasites cause few symptoms. *D. immitis* infections in particular are therefore frequently detected only incidentally and are initially often confused with malignancies.

*D. repens* has been reported from various regions of the world with warm to moderate climate, but only very rarely from Romania [1, 4]. We report a human *D. repens* infection from Romania, which produced an atypical and severe clinical picture.

## 2. Case Presentation

A male, 31-years-old patient, with no significant medical history, presented in February 2011 with a cellulitis-like plaque on the right inner thigh (10 cm diameter) that, in about 12 hours, extended to almost the entire thigh. He was living in a Romanian suburban area (near Bucharest) and had a pet dog. The patient denied recent or past travel abroad, except for France (Paris) for a weekend, 4 months earlier (in October). The putative diagnosis at that time was cellulitis, and antibiotic treatment was started with amoxicillin-clavulanic acid (for 5 days). All symptoms subsided rapidly within 24–48 hours.

One week later the patient developed severe muscle pain in the lower right leg, and after a further two days he noticed a similar cellulitis-like plaque as before (red, edematous, warm with imprecise limits) in the popliteal region, with rapid extension to the adjacent areas, associated with impaired walking ability due to extensive edema. Doppler ultrasound showed no signs of thrombosis in the right leg. Laboratory investigations showed mild eosinophilia (7.2%; normal range 0–7%). All other investigations including complete blood count, sedimentation rate, urinalysis, and liver and renal function tests were normal. Because of the severity of symptoms, treatment with the macrolide antibiotic azithromycin and the low-molecular-weight heparin enoxaparin was administered for 7 days. Again the symptoms resolved quickly and completely.

**Figure 1:**
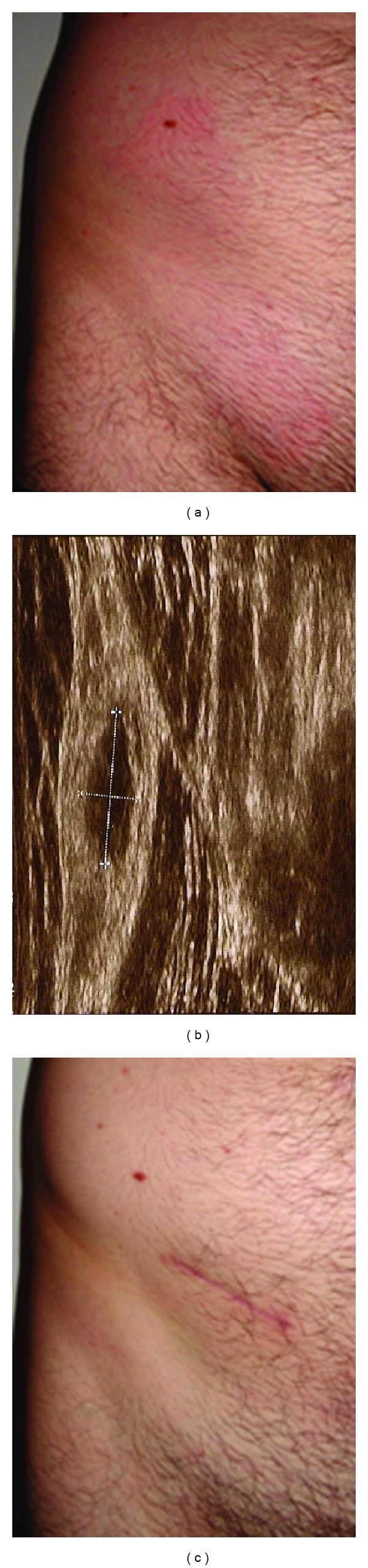
Clinical picture before and after surgery. Before the removal of the parasite, the patient showed erythematous plaques with mild edema in the lower right abdominal region (a). The ultrasound revealed a well-defined cystic lesion of 11.9 mm × 4.8 mm above muscular layer, which is seen on the right (b). After excision of the nodule the wound healed without complications and all symptoms resolved. The scar reveals the final location of the parasite (c).

After about two further weeks, the patient developed similar but less severe lesions on the lower right part of the abdomen that were again associated with muscle pain (Figure 1(a)).

Infectious disease screening was performed and showed positive serology for *Echinococcus (E.) granulosus *IgG (ELISA) and negative tests for *Borrelia burgdorferi*, *Toxoplasma gondii*, *Toxocara canis*, *Trichinella spiralis*, EBV, CMV, HBV, HCV, and HIV. Abdominal ultrasound and chest radiography were normal.

Because of the eosinophilia and the positive *E. granulosus* serology, a parasitological cause was suspected and albendazole treatment was started (800 mg/day). During antiparasitic therapy, the inflammatory abdominal plaque resolved, but a nodule developed deep in the subcutaneous tissue (Figure 1(b)). The nodule was excised, and all signs of infection including the eosinophilia subsided, and no further symptoms developed (Figure 1(c)).

**Figure 2:**
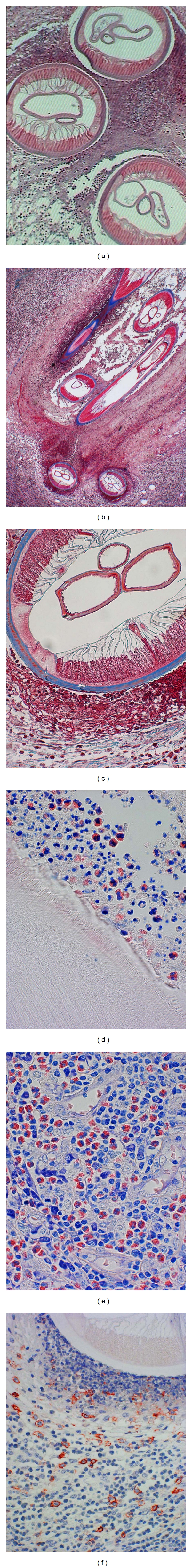
Histological sections of the parasite. Sections were stained using hematoxylin-eosin (HE) (a), Masson's trichrome stain (b, c), Giemsa (d, e), and periodic acid-Schiff reaction and immunohistochemistry for IgE (f). Typical features of *D. repens* are the external longitudinal ridges, the thick cuticula, and the well-developed musculature (a, b, c). Around the parasite a marked inflammatory reaction with numerous eosinophils, neutrophils, lymphocytes, plasma cells, and macrophages is seen (d, e). Many IgE-positive plasma cells are present in the infiltration and near to the surface of the parasite (f).

The excised material was investigated histopathologically using hematoxylin-eosin (HE), Giemsa, and Masson's trichrome stains, periodic acid-Schiff reaction, and immunohistochemistry for IgE (Figure 2). The histopathology was similar to that described for subcutaneous dirofilariasis in previous reports [5, 6]. The parasite showed the typical features of a nongravid female *D. repens*. The most indicative structures were the external longitudinal ridges, leading to a cogwheel-like appearance in the transverse sections (Figures 2(a), 2(b), and 2(c)). Further typical features were the thick cuticula and the well-developed musculature (Figures 2(a) and 2(c)). Around the parasite a marked inflammatory reaction had developed (Figures 2(d) and 2(e)). The histological slides showed numerous eosinophils, neutrophils, lymphocytes, plasma cells, and macrophages. (Figures 2(d) and 2(e)). Occasionally, eosinophils and neutrophils, partly with pycnotic nuclei, were seen on the surface of the parasite (Figures 2(d) and 2(e)). Many IgE-positive plasma cells were present in the infiltration and near to the surface of the parasite (Figure 2(f)).

To confirm the morphological diagnosis, DNA was isolated from the tissue sample embedded in paraffin wax and a PCR targeting the mitochondrial 12S rRNA was performed as described before [7, 8]. Sequence analysis of the PCR product showed the highest similarity to Italian isolates of *D. repens*, thus confirming the morphological identification of our isolate as *D. repens.*

Serum samples were investigated retrospectively for antifilarial antibodies using an ELISA composed of *D. immitis* antigen. The test was positive as expected. In addition, the presence of antibodies against *E. granulosus* was confirmed by ELISA and indirect hemagglutination, but not by a western blot confirmation test. The patient was thoroughly screened for signs of an *Echinococcus* infection by sonography and CT scans, but no indications of a hydatid cyst were found.

In order to exclude the patient's dog as a source of (further) *D. repens* infections, blood samples were obtained and investigated for microfilaria using Giemsa stain and PCR. No microfilaria or filarial DNA was found.

## 3. Discussion

We present a case of human *D. repens* infection with an atypical presentation that was most likely acquired in Romania. Human infection with *D. repens* usually presents as larva migrans and later in the course of infection as a subcutaneous nodule [2, 3]. However, infection may cause a wide variety of symptoms depending on the site of infection [2, 3]. In our case *D. repens* infection was associated with an unusual clinical presentation, namely, signs of cellulitis and deep venous thrombosis. We cannot definitively say what caused the symptoms in our case and cannot exclude any cause of the symptoms other than *D. repens.* It seems extremely unlikely that an *Echinococcus* infection was responsible for the symptoms since we did not find any sign of *Echinococcus* infection. It could not be clarified whether the positive reactions with the *E. granulosus* tests were caused by an asymptomatic previous contact with *Echinococcus* or just by unspecific cross-reactions. The finding of *Echinococcus* antibodies with ELISA must be interpreted as an incidental finding without relevance for the described case.

The facts that the symptoms developed close to the site of the *D. repens* worm and that all symptoms subsided only when the *D. repens* was removed strongly suggest that the worm was the true cause of the symptoms. Most probably the worm initially wandered in the subcutaneous tissue and provoked a local immune response. It may be speculated that the antibiotic treatment contributed to the temporary resolution of symptoms. A possible mechanism would be by attack on the endosymbiont *Wolbachia*, which contributes to the immune reaction of the host [9]. Equally, the worm may have wandered to another less sensitive place, and the temporary resolution of symptoms after antibiotic treatment might have been coincidental. However, the antiparasitic treatment most likely had an effect and indeed led to the arrest of the worm, allowing its subsequent removal. Because usually only one infertile parasite is present, removal of the worm is sufficient. However, it is scarcely possible to locate and surgically remove a subcutaneously wandering worm, because the main signs of infection are present in the region that the parasite has just vacated. As long as the worm is not wandering through the subconjunctiva, where it can be precisely located, surgery should not be performed before the worm has stopped its journeying. As our case shows, an anthelmintic treatment can be used to stop the worm. Such anthelmintic therapy leading to the arrest of a wandering worm and allowing removal of the worm has successfully been applied before (G. Just-Nuebling, Frankfurt, personal communication, 2011).

The exact source of infection of our patient could not be determined. We did not find signs of infection with *D. repens* in the patient's dog. Considering the longevity of the worm in its final host, the dog, it is unlikely but not impossible that the patient's dog was the source of its owner's infection. It is highly unlikely that the patient acquired the infection during the short trip to Paris because the disease is hardly present in French urban dogs and there are only very low numbers of mosquitoes in Paris in October. The infection was therefore most likely acquired in the region of residence (near Bucharest) in Romania. Only a few cases of human infection with *D. repens *have so-far been described in Romania [4]. It is well known that *D. repens* occurs in moderate climatic zones of Europe, with a hot spot in northern Italy [2, 3]. Recently, increasing numbers of human dirofilariasis cases have been reported from France and Greece, and first cases have even been found north of the Alps, in countries such as the Czech Republic, Hungary, Russia, and Austria [1, 10–13]. It has been suggested that *D. repens* is an emerging disease that is spreading under the influence of global climate warming [14]. *D. repens* is thus probably more frequent in Romania than the so-far sparse case reports imply, and the number of cases might be increasing.

Human doctors as well as veterinarians should therefore be prepared to come across cases of dirofilariasis in Romania, as well as in other European countries regarded nonendemic so far. As our case illustrates, infection should even be considered in patients with signs of parasitic infection that do not exactly correspond to the description of dirofilariasis in the text books.
